# Clostridioides difficile infections in seven Brazilian hospitals during the COVID-19 pandemic: hand hygiene and antimicrobial consumption

**DOI:** 10.1590/S1678-9946202668031

**Published:** 2026-05-18

**Authors:** Luiza Arcas Gonçalves, Ivan Lira dos Santos, Ana Paula Matos Porto, Antônio Brazil Viana, Julia Herkenhoff Carijo, Claudia Dantas de Maio Carillho, Brunno Cesar Batista Cocentino, Luciana Neves Passos, Glaucia Fernanda Varkulja, Thais Guimarães, Silvia Figueiredo Costa

**Affiliations:** 1 Universidade de São Paulo, Faculdade de Medicina, Hospital das Clínicas, São Paulo, São Paulo, Brazil; 2 Pontifícia Universidade Católica de Campinas, Campinas, São Paulo, Brazil; 3 Universidade de São Paulo, Faculdade de Medicina, Instituto de Medicina Tropical de São Paulo (LIM-49), São Paulo, São Paulo, Brazil; 4 Universidade de São Paulo, Faculdade de Medicina, Departamento de Infectologia e Medicina Tropical, São Paulo, São Paulo, Brazil; 5 Centers for Antimicrobial Optimization Network, São Paulo, São Paulo, Brazil; 6 Universidade Federal do Ceará, Fortaleza, Ceará, Brazil; 7 Hospital Glória D’Or, Rio de Janeiro, Rio de Janeiro, Brazil; 8 Universidade Estadual de Londrina, Londrina, Paraná, Brazil; 9 Hospital Paulistano, São Paulo, São Paulo, Brazil; 10 Hospital Unimed, Espírito Santo, Brazil; 11 Hospital Santa Catarina, São Paulo, São Paulo, Brazil; 12 Hospital do Servidor Público Estadual, São Paulo, São Paulo, Brazil

**Keywords:** Clostridioides difficile, COVID-19, Antibiotic consumption

## Abstract

This study aimed to evaluate the incidence density of *Clostridioides difficile* infections (CDIs), hand hygiene adherence, and antimicrobial consumption across Brazilian hospitals during the COVID-19 pandemic. An ecological study was conducted in seven Brazilian hospitals from June 2018 to December 2019 (pre-pandemic) and from June 2020 to December 2021(pandemic). Data on CDI incidence density, hand hygiene adherence, and antimicrobial consumption were collected. CDI incidence was assessed based on the number of monthly cases and the incidence density rate per 10,000 patient-days. This study involved hospitals from four states and included three public and four private institutions. Statistical analyses were performed using the Mann-Whitney U test in R (*p* < 0.05). Time series analysis used joinpoint regression to determine monthly percent change, average monthly percent change, and 95% confidence intervals. Although statistically insignificant, an upward trend occurred in CDI incidence (*p* = 0.081) from the pre-pandemic to the pandemic periods. Consumption of azithromycin (*p* < 0.01) and levofloxacin (*p* < 0.01) increased significantly, whereas ceftriaxone use decreased (*p* = 0.0038). The joinpoint regression analysis of antimicrobial consumption trends during the pandemic showed trend changes for vancomycin and meropenem defined daily dose, with higher consumption during the second wave of COVID-19 in Brazil. Hand hygiene adherence failed to differ significantly but showed a downward trend in 2020 and 2021. The dynamics of the COVID-19 pandemic and changes in protective measures may have influenced CDI incidence and antimicrobial consumption patterns across the study period.

## INTRODUCTION

Despite the global nature of the COVID-19 pandemic, its impact varied significantly across countries. In Latin America, densely populated municipalities, previously strained health systems, and the limited availability of diagnostic tests resulted in some of the most severe health challenges of the century. This region struggled to control virus transmission and to provide adequate health care^1^. Brazil, in particular, stood among the countries that were hit the hardest by the pandemic^2^. It was the second most affected country globally, accounting for 5.48% of all infections and over 696,000 deaths, representing 10.21% of the all deaths worldwide^2^.

To mitigate the effects of the virus, Brazil significantly restructured its health system, which included increasing the number of intensive care unit (ICU) beds, hiring more healthcare providers, and optimizing state and municipal health transfers^2^. Despite these efforts, the Brazilian health system, particularly its public Unified Health System (SUS), which serves 75% of its population, faced challenges such as overcrowding, overburdened healthcare providers, and shortages of essential supplies, including personal protective equipment (PPE)^3^, oxygen, and mechanical ventilators^4^. Such high demand and inadequate care may have contributed to an increase in healthcare-associated infections, including those of *Clostridioides difficile* (CDI)^5^.

The impact of the COVID-19 pandemic on CDI requires further investigation, particularly in Latin America and Brazil^6-9^. The country shows a notable lack of epidemiological data on CDIs^10,11^ before and during the pandemic. This raises questions about how the pandemic may have influenced the incidence of CDI in public and private hospitals in Brazil.

This study aimed to assess the incidence density of CDIs during the COVID-19 pandemic and the preceding period in seven Brazilian hospitals and to evaluate hand hygiene compliance and antimicrobial consumption rates.

## MATERIALS AND METHODS

### Ethics

This project was approved by the Research Ethics Committee of the University of Sao Paulo, School of Medicine, Hospital das Clinicas on November 11, 2022 (CAAE nº 62937022.0.0000.0068).

### Study design

This retrospective, observational, ecological, and multicenter study was carried out in seven Brazilian hospitals in four states (Sao Paulo, Espirito Santo, Rio de Janeiro, and Parana) that treated COVID-19 patients from June 2020 to December 2021. Data were collected for two periods: pre-pandemic (June 2018 to December 2019) and pandemic (June 2020 to December 2021) to evaluate CDI incidence, hand hygiene compliance, and antimicrobial consumption. Data collection involved the submission of standardized information from each hospital to the principal investigator for analysis. Data from March to May 2020 were excluded due to the initial disruption of infection prevention and control (IPC) committee activities at the onset of the COVID-19 pandemic, which resulted in insufficient data for reliable analysis.

CDI incidence was assessed by the absolute number of monthly cases and the CDI density rate per 10,000 patient-days for each hospital^12^. Cases were individually reviewed by the infection control committee of each hospital based on clinical criteria (unexplained and new-onset ≥3 unformed stools in 24 h), length of stay (>72 h), and positive laboratory tests [e.g., enzyme immunoassay for toxins A and B, glutamate dehydrogenase (GDH) assay, *C. difficile* DNA polymerase chain reaction, and/or immunochromatographic tests detecting GDH antigen and toxins A/B simultaneously]. According to laboratory-reported specifications of the immunochromatographic assay, GDH sensitivity and specificity total 98.1% and 90.7% and toxins A/B sensitivity and specificity, 65.0% and 97.0%, respectively^13,14^. Hand hygiene compliance was evaluated by the percentage of appropriate hand hygiene opportunities according to the five moments recommended by the World Health Organization: before patient contact, before aseptic procedures, after exposure to body fluids, after patient contact, and after touching nearby surfaces^15^. Only direct observation assessments, conducted as part of routine IPC committee activities in each institution by cross-sectional monthly observations, were included. Hand hygiene data based on alcohol-based hand rub consumption were ignored in the analysis.

Antimicrobial consumption was measured using the defined daily dose (DDD) for intravenous formulations of clindamycin, azithromycin, ceftriaxone, levofloxacin, meropenem, piperacillin-tazobactam, and vancomycin. DDD was defined as the average daily dose used by an adult for the main therapeutic indication. It was calculated by dividing the total amount of antimicrobial consumed (in grams) by the standard daily adult dose (70 kg, without renal failure), then dividing it by the number of patient-days in the evaluation period^16^. Additionally, data on the number of ward and ICU beds, patient-days, monthly hospital and ICU admissions, and COVID-19 admissions (both laboratory-confirmed and clinical radiological diagnoses) were collected. The proportion of ICU admissions relative to total hospital admissions and the proportion of COVID-19-related admissions were also evaluated. Information on antimicrobial stewardship programs, including team composition, antibiotic control strategies, and operations during the pandemic, was also gathered. Hospitals were categorized as public or private.

### Statistical analysis

Categorical variables are shown as absolute numbers and percentages, whereas continuous ones, as medians with interquartile ranges. Comparisons of patient-days, total and ICU admissions, antimicrobial stewardship program operations, hand hygiene compliance rates, antimicrobial consumption (expressed as DDD), and CDI incidence density before the pandemic (June 2018 to December 2019) and during it (June 2020 to December 2021) were performed by the Mann-Whitney U test. Differences in the proportion of COVID-19 admissions across the seven centers were evaluated using the Pearson’s chi-squared test with pairwise comparisons that were adjusted by the Bonferroni correction. Differences in the overall proportion of ICU admissions between the pre-pandemic and pandemic periods were evaluated by a two-proportion z-test based on aggregated hospital data. Data analysis was conducted on R, version 4.1.0. A p-value <0.05 was considered statistically significant.

A time-series analysis of antimicrobial DDD during the pandemic was conducted, along with a comparison of CDI incidence density with COVID-19 admissions (including the proportion of COVID-19 admissions and total patient numbers) and DDD for meropenem, ceftriaxone, and levofloxacin. These antimicrobials were selected based on data availability across all centers and their established association with CDI risk. CDI incidence density was further analyzed using joinpoint segmented regression on the Joinpoint Regression Program (version 5.0; Statistical Research and Applications Branch, Surveillance Research Program, National Cancer Institute, Bethesda, Maryland, USA), estimating the monthly percentage change and the average monthly percentage change with 95% confidence intervals (95%CI). The number of joinpoints for each segment was automatically selected using the default settings of the software, and a logarithmic transformation of the outcome variables was applied prior to analysis.

## RESULTS

This study evaluated data from seven hospital centers: six provided data from June 2018 to December 2019 (pre-pandemic) and from June 2020 to December 2021 (pandemic), whereas one only provided data for the pandemic. This sample included four private and three public hospitals, all classified as high complexity. Among the public hospitals, two were very large (over 500 beds) and one was large (151 to 500 beds). Among the private hospitals, two were large and two were medium-sized (51 to 150 beds).

Regarding *C. difficile* diagnosis, five institutions used simultaneous tests on fecal samples, with four employing immunochromatographic tests for toxins A and B and GDH and one using both tests separately. One center performed sequential testing with ELISA for toxins A and B followed by polymerase chain reaction for *C. difficile* DNA, whereas another used ELISA for toxins A and B alone. No hospital altered or suspended its diagnostic methods during the study period.

Regarding the antimicrobial stewardship program, six of the seven hospitals had formal programs. In total, three programs were led solely by infectious disease physicians, whereas the others included clinical pharmacists as part of their multidisciplinary teams. Overall, five hospitals used pre-authorization strategies for selected antimicrobials, and all hospitals conducted post-prescription audits, with one hospital performing these audits retrospectively. Only one hospital suspended its stewardship activities during the COVID-19 pandemic.

In the aggregate analysis, all hospitals showed low CDI incidence densities, with a median of less than two cases per 10,000 patient-days, except for one private hospital, which had a median of 5.17 and an interquartile range up to 8.22. This study evaluated hand hygiene compliance in five hospitals, with three private hospitals achieving over 75% adequacy, whereas one private and one public hospital had rates below 65%, with the public hospital showing the lowest compliance. This research collected antimicrobial consumption data for meropenem, ceftriaxone, levofloxacin, piperacillin-tazobactam, and vancomycin from all hospitals. Also, two hospitals also provided data for azithromycin and clindamycin. Table 1 shows detailed data from each hospital.

**Table 1:** Characteristics of the seven hospitals regarding CDI incidence, admissions, patient-days, hand hygiene compliance, and antimicrobial consumption in seven Brazilian hospitals

	A (N=19^a^)	B (N=38^a^)	C (N=38^a^)	D (N=38^a^)	E (N=38^a^)	F (N=38^a^)	G (N=38^a^)
**CDI incidence density** (per 10.000 patient-day)	0.00 (0.00–2.62)	1.17 (0.56–2.33)	1.58 (1.32–2.52)	0.00 (0.00–2.78)	1.45 (0.00–2.78)	0.00 (0.00–1.22)	5.17 (2.41–8.22)
**CDI cases**	0.00 (0.00–0.50)	2.00 (1.00–4.00)	3.00 (2.00–4.00)	0.00 (0.00–1.00)	1.00 (0.00–2.00)	0.00 (0.00–1.00)	2.00 (1.00–3.00)
**Hospital beds**	97 (97–97)	874 (795–910)	714 (712–715)	145 (145–145)	316 (316–342)	378 (363–393)	184 (184–184)
**Total admissions**	472 (373–504)	2,380 (1,686–2,707)	2,094 (1,884–2,650)	902 (808–949)	2,558 (1,860–3,411)	1,429 (1,306–1,489)	1,464 (1,279–1,668)
**ICU admissions**	114 (94–159)	393 (360–502)	358 (285–424)	134 (114–158)	1,524 (1,440–1,714)	243 (128–290)	256 (236–286)
**COVID-19 admissions**	29 (13–62)	520 (270–752)	233 (207–233)	10 (5–19)	215 (90–260)	393 (265–486)	111 (56–185)
**Hospital patient-days**	1,688 (1,222–1,948)	16,714 (14,206–17,775)	15,808 (14,825–18,090)	3,577 (3,429–3,810)	7,152 (6,846–7,607)	8,440 (7,795–9,504)	4,201 (3,904–4,445)
**ICU patient-days**	795 (568–988)	2,878 (2,760–3,349)	1,570 (1,476–1,732)	452 (411–495)	1,898 (1,774–2,038)	783 (747–2,083)	1,220 (1,087–1,310)
**Hand hygiene compliance**	0.61 (0.56–0.74)	0.55 (0.43–0.65)	NA (NA–NA)	0.84 (0.83–0.86)	0.83 (0.77–0.86)	NA (NA–NA)	0.77 (0.62–0.84)
**DDD** (g per 1,000 patient-days)							
Clindamycin	NA (NA–NA)	NA (NA–NA)	NA (NA–NA)	NA (NA–NA)	13 (9–21)	NA (NA–NA)	16 (13–22)
Azithromycin	NA (NA–NA)	NA (NA–NA)	NA (NA–NA)	NA (NA–NA)	2 (0–7)	NA (NA–NA)	14 (8–35)
Ceftriaxone	60 (39–72)	116 (80–144)	142 (118–180)	174 (151–214)	134 (110–166)	90 (72–122)	114 (95–134)
Levofloxacin	76 (39–95)	17 (10–24)	3 (0–19)	4 (0–12)	13 (6–20)	62 (47–86)	8 (0–30)
Meropenem	231 (199–280)	132 (110–155)	223 (179–290)	108 (81–146)	118 (103–152)	382 (301–432)	148 (132–188)
Piperacilin-tazobactam	124 (103–170)	62 (43–83)	158 (109–174)	215 (174–275)	64 (56–77)	155 (114–194)	78 (58–104)
Vancomycin	54 (38–80)	93 (82–117)	208 (189–241)	87 (74–111)	38 (30–45)	223 (180–274)	92 (70–102)
**Stewardship program**							
Yes	19 (100%)	38 (100%)	38 (100%)	38 (100%)	19 (50%)	38 (100%)	0 (0%)
No	0 (0%)	0 (0%)	0 (0%)	0 (0%)	19 (50%)	0 (0%)	38 (100%)
**Period**							
Pre-pandemic	0 (0%)	19 (50%)	19 (50%)	19 (50%)	19 (50%)	19 (50%)	19 (50%)
Pandemic	19 (100%)	19 (50%)	19 (50%)	19 (50%)	19 (50%)	19 (50%)	19 (50%)

^a^Median (IQR); n (%); CDI = *Clostridioides difficile* infection; ICU = Intensive care unit; DDD = Defined daily doses.

This study compared the pandemic and the pre-pandemic periods among the six hospitals that provided data for both timeframes (Table 2). This study found no statistically significant difference in the incidence density of CDI between the two periods, although the pandemic showed a higher median. This result was consistent across the institution types. Total hospital admissions were significantly lower during the pandemic, and patient-days showed a statistically significant reduction in 2020 and 2021. In the aggregated analysis, the proportion of ICU admissions differed between periods, with a significant increase of 5.40% during the pandemic (*p* < 0.0001; OR=1.488).

**Table 2:** Comparison of CDI incidence, stewardship program, antimicrobial consumption, and hand hygiene adherence between the pre-pandemic and the COVID-19 pandemic periods in six Brazilian hospitals.

	COVID-19 Pandemic		
	No	Yes	p-value^b^
N	**114** ^**a**^	**114** ^**a**^	
**CDI incidence**			
Total	1.40 (0.00–2.71)	1.74 (0.00–3.05)	0.2
Public	1.15 (0,00–2.14)	1.35 (0.57–2.18)	0.3
Private	1.57 (0.00–3.14)	2.69 (0.00–4.08)	0.4
**Patients-days**	7456 (4520–17106)	7849 (3904–13918)	**0.037**
**Admissions**			
Total	2040 (1422–2762)	1528 (1167–1830)	**<0.001**
ICU	356 (196–440)	287 (226–502)	0.7
***Stewardship program***			**0.004**
No	19 (17%)	38 (33%)	
Yes	95 (83%)	76 (67%)	
Hand hygiene	0.78 (0.52–0.85)	0.65 (0.56–0.78)	0.084
**DDD**			
Clindamycin	16 (11–26)	13 (9–18)	0.068
**Azithromycin**	0 (0–2)	8 (5–20)	**<0.001**
**Ceftriaxone**	138 (103–166)	123 (92–152)	**0.0038**
**Levofloxacin**	9 (1–20)	19 (8–46)	**<0.001**
Meropenem	152 (118–192)	168 (109–271)	0.6
Piperacillin-tazobactam	88 (60–205)	105 (78–144)	0.6
Vancomycin	91 (63–188)	114 (71–185)	0.3

^a^Median (IQR); n (%); ^b^Mann-Whitney U test; Pearson’s chi-squared test; CDI =: *Clostridioides difficile* infection; ICU = Intensive care unit; DDD = Defined daily doses.

Hand hygiene compliance rates showed no statistically significant difference between the periods, with the lowest median observed from June 2020 to December 2021. However, antimicrobial stewardship program operation showed differences as one hospital suspended its activities during the COVID-19 pandemic. Antimicrobial consumption (expressed as DDD) varied between the periods; azithromycin and levofloxacin consumption increased during the pandemic, whereas ceftriaxone consumption decreased (Table 2). No significant changes occurred in the consumption of clindamycin, meropenem, piperacillin-tazobactam, or vancomycin.

**Figure 1:**
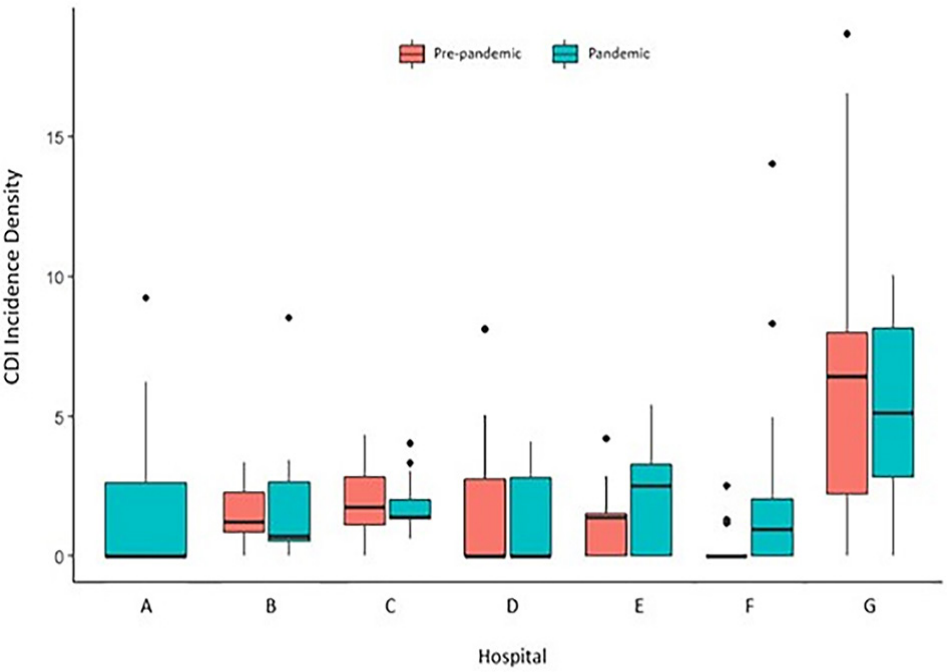
Pre-pandemic and pandemic CDI incidence density in seven Brazilian hospitals.

Figure 1 shows the comparison of CDI incidence density between the two periods, stratified by hospital center. Hospital G showed higher CDI incidence in both periods, although its median decreased during the pandemic. Hospitals E and F experienced an increase in incidence during the pandemic, whereas Hospital D showed stable incidence rates. Conversely, Hospitals B and C showed a reduction in median CDI incidence during the pandemic. Except for Hospital A, which provided data only for the pandemic, graphical analysis shows the differences in baseline median CDI incidence density among centers during the pre-pandemic.

The evaluation of the pandemic (June 2020 to December 2021) included data from all seven hospitals. The assessment of the proportion of COVID-19 admissions across centers showed statistically significant differences between the seven hospitals (*p* < 0.001). Hospital F had the highest proportion of COVID-19 hospitalizations (27.6%), followed by Hospital B (20.5%). The remaining hospitals showed lower proportions: Hospital C (11.6%), Hospital E (10.0%), Hospital A (9.4%), Hospital G (9.0%), and Hospital D (1.6%), as illustrated in Supplementary Figure S1.

**Figure 2:**
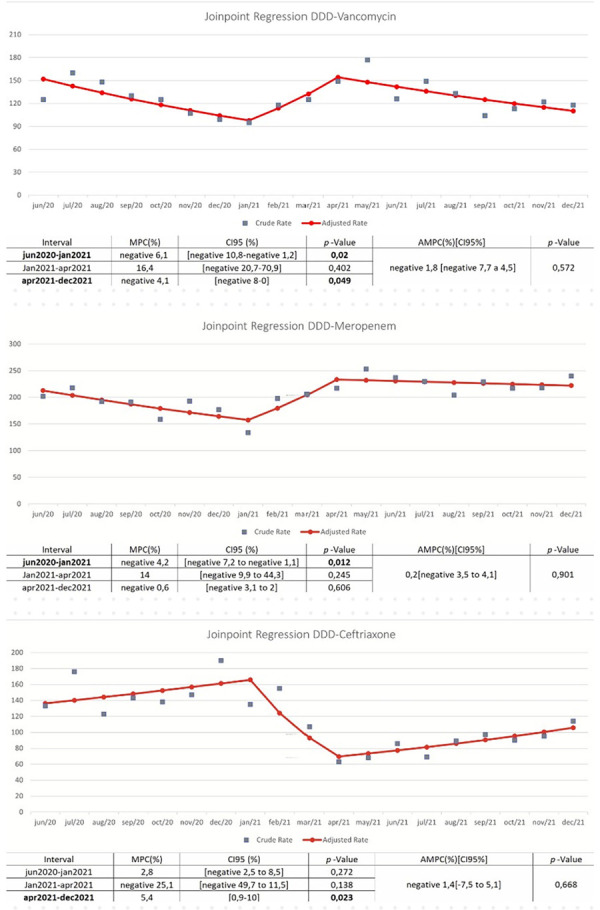
Joinpoint regression of vancomycin, meropenem, and ceftriaxone DDD in seven Brazilian Hospitals, from 2020 to 2021.

To analyze vancomycin, ceftriaxone, and meropenem consumption, this study evaluated time series data from June 2020 to December 2021 using joinpoint regression (Figure 2). Vancomycin DDD showed an average percentage reduction of 1.8% over the period, although this change was statistically insignificant. However, significant reductions occurred: 6.1% from June 2020 to January 2021 and 4.1% from April to December 2021 (*p* < 0.05). Ceftriaxone consumption showed an average percentage reduction of 1.4% over the entire period (*p* = 0.668) but a significant increase of 5.4% from April to December 2021. Conversely, meropenem DDD showed a numerical increase of 0.2% over the entire period, although this change was statistically insignificant (*p* = 0.091). Joinpoint analysis found a statistically significant reduction of 4.2% from June 2020 to January 2021.

**Figure 3:**
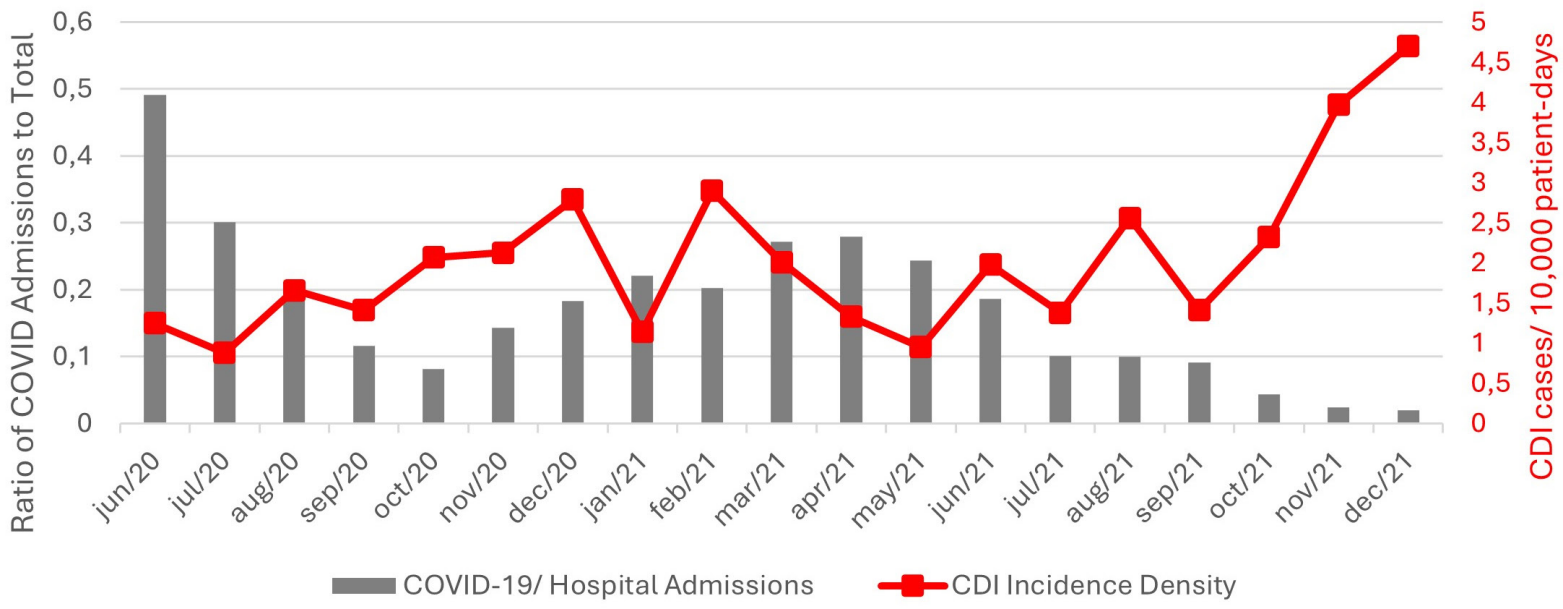
Trends in CDI incidence density and COVID-19 admissions across seven Brazilian hospitals.

This study also performed a descriptive time-series analysis of the relationship between COVID-19 admissions, total admissions, and CDI incidence density during the pandemic period. It observed a higher proportion of COVID-19 admissions in June 2020 and in March to June 2021, corresponding to the peaks of the first and second waves of SARS-CoV-2 cases in Brazil. CDI incidence density showed no corresponding increase during these months but exhibited an upward trend from September 2021, coinciding with a significant reduction in the proportion of COVID-19 admissions, as illustrated in Figure 3.

**Figure 4:**
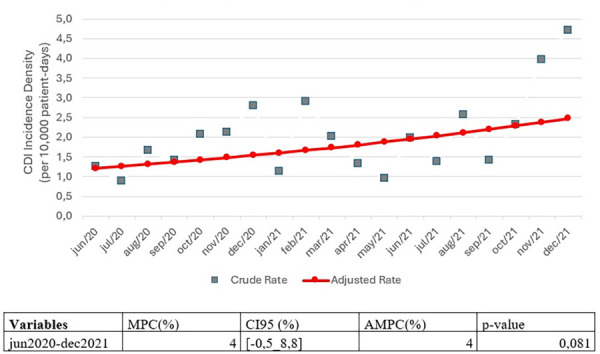
Joinpoint regression of *Clostridioides difficile* infection (CDI) incidence density in seven Brazilian hospitals during the COVID-19 pandemic

Joinpoint regression was performed to analyze the temporal trend of CDI incidence density, as illustrated in Figure 4. This study found no statistically significant distinctions in line segments and no significant inflection point (*p* = 0.081). However, it observed a mean percentage increase of 4% (95%CI −0.5-8.8) over the entire period and a 4% increase between years based on the adjusted rates.

## DISCUSSION

The impact of COVID-19 pandemic on CDI remains controversial in data from the U.S., Canada, and Europe^5,6,8,9,17-21^, with only one study from Latin America reporting a reduction in CDI incidence during the pandemic^22^. In this multicenter study, CDI incidence showed a non-significant upward trend during the pandemic. This research also found a decline in ceftriaxone consumption during the pandemic, followed by significant increase in the use of azithromycin and levofloxacin.

A pandemic caused by a virus transmitted via airborne and contact routes^23^ required universal precautions, including contact and droplet measures, and aerosol measures for COVID-19 patients^24^. Measures reinforced environmental cleaning and hand hygiene, along with the routine use of gowns and gloves during patient care. Given the importance of spore transmission in CDI, these infection control measures could have theoretically reduced CDI incidence.

The use of gloves may also have contributed to the control of CDI transmission^12^ as COVID-19 patients routinely placed under contact isolation with universal PPE recommendations. However, adherence to PPE varied widely across settings, ranging from 85% in Germany^25^, 90% in Ghana^26^, and 60% in the United States^27^. In Brazil, PPE adherence in 2020 reached approximately 70%^28^, further challenged by periods of limited equipment availability^3^.

Several studies have reported a significant increase in hand hygiene adherence during the pandemic^29,30^, particularly during the early waves of infection^31,32^. In contrast, our study observed no statistically significant differences in hand hygiene adherence between the pandemic and the pre-pandemic periods, although it found a downward trend throughout 2020 and 2021. Data on the impact of the pandemic on environmental hygiene remain limited, highlighting important gaps in the current literature. Therefore, this aspect was not assessed in the present study.

Conversely, the increased use of antimicrobials^33,34^—particularly broad-spectrum agents^35,36^—may have contributed to higher rates of CDI rates, especially in intensive care units. Prolonged hospitalization of older patients with multiple comorbidities^20^, particularly during the first wave of COVID-19, represents an additional non-modifiable risk factor for CDI.

This study observed an increase in azithromycin and levofloxacin during the pandemic when compared to the pre-pandemic period and a decrease in ceftriaxone consumption. We found no statistically significant changes in the consumption of meropenem, vancomycin, or piperacillin-tazobactam, consistent with previous Brazilian findings^7,37^. Although macrolides are generally considered to pose a lower risk for *C. difficile* infection, fluoroquinolones are strongly associated with CDI, as are cephalosporins^38,39^. Despite these findings, this study observed no statistically significant difference in CDI incidence density between the two periods.

Analysis of the historical series during the pandemic showed an upward trend in the incidence of CDI, albeit statistically insignificant, which may have been influenced by changes in testing frequency. Notably, CDI incidence density increased more markedly from September 2021 onward, coinciding with a decline in the number of hospitalized COVID-19 cases. Conversely, antimicrobial consumption did not show a corresponding decline, with an increased DDD of meropenem occurring in the final months of the pandemic. Joinpoint regression analysis found trend changes in vancomycin and meropenem DDD, with higher consumption during the second wave of COVID-19 in Brazil (November 2020 to April 2021).

Although no participating hospital halted laboratory testing for *C. difficile* during 2020 and 2021, CDI may have been underdiagnosed during this period. A global reduction in testing when compared with the pre-pandemic era has been reported, particularly given the overlap between gastrointestinal symptoms of SARS-CoV-2 infection and CDI and the reluctance to handle secretions from COVID-19-positive patients^40^. Since the number of performed CDI tests was not quantified in this study, we were unable to formally assess this hypothesis, potentially limiting comparisons between the two periods.

### Limitations

As this was a retrospective study, complete standardization of diagnostic methods across centers was not possible. One participating hospital relied exclusively on ELISA for toxins A and B, a method with lower diagnostic sensitivity, which may have led to CDI case underestimation. Moreover, this study reported data on hand hygiene adherence according to the routine auditing practices of the IPC committee of each hospital, which may have introduced variability in measurement across institutions. Institutions also showed heterogeneity regarding the proportion of hospitalizations attributable to COVID-19.

Despite the increase in the proportion of ICU admissions during the pandemic, inferences regarding individual clinical severity were impossible to the ecological study design of this research. Furthermore, differences in ICU admission thresholds across participating centers limit the use of ICU utilization as a proxy for disease severity.

Additional limitations include the relatively small number of participating hospitals, which may reduce statistical power. The use of non-parametric tests on correlated data represents an additional limitation since monthly observations within the same hospital are not independent. However, the inclusion of large, high-complexity centers enabled the analysis of a substantial number of patient-days. Despite these limitations, this study provides important multicenter data from Brazil, a region with limited epidemiological information on CDI.

## CONCLUSIONS

The dynamics of the COVID-19 pandemic and changes in protective measures may have influenced CDI in different ways. Although this study observed no statistically significant difference in CDI incidence between the pre-pandemic and pandemic periods, it found a non-significant upward trend in 2020 and 2021. Antimicrobial consumption patterns also changed, with a decline in ceftriaxone use and significant increases in azithromycin and levofloxacin consumption during the pandemic.

## Supplementary Materials

SUPPLEMENTARY MATERIAL

## Data Availability

The complete anonymized dataset supporting the findings of this study is available from https://doi.org/10.48331/SCIELODATA.QHC4EB
