# Supplementary material for: Clostridioides difficile infections in seven Brazilian hospitals during the COVID-19 pandemic: hand hygiene and antimicrobial consumption
Source: Rev Inst Med Trop Sao Paulo. 2026 May 18;68:e31. doi: 10.1590/S1678-9946202668031 (PMC13185524; doi:10.1590/S1678-9946202668031)
Supplement: SUPPLEMENTARY MATERIAL [file 1678-9946-rimtsp-68-S1678-9946202668031-suppl01.pdf]

REVISTA DO INSTITUTO DE MEDICINA TROPICAL DE SÃO PAULO

JOURNAL OF THE SÃO PAULO INSTITUTE OF TROPICAL MEDICINE

SUPPLEMENTARY MATERIAL

https://doi.org/10.48331/SCIELODATA.QHC4EB

Clostridioides difficile infections in seven Brazilian hospitals during the COVID-19 pandemic: hand hygiene and antimicrobial consumption

Luiza Arcas Gonçalves [1], Ivan Lira dos Santos [2], Ana Paula Matos Porto [3,4,5], Antônio Brazil Viana Junior [6], Julia Herkenhoff Carijo [7], Claudia Dantas de Maio Carillho [8], Brunno Cesar Batista Cocentino [9], Luciana Neves Passos[10], Glaucia Fernanda Varkulja [11], Thais Guimarães [1,12], Silvia Figueiredo Costa [1,3,4]

[1]Universidade de São Paulo, Faculdade de Medicina, Hospital das Clínicas, São Paulo, São Paulo, Brazil

[2]Pontifícia Universidade Católica de Campinas, Campinas, São Paulo, Brazil

[3]Universidade de São Paulo, Faculdade de Medicina, Instituto de Medicina Tropical de São Paulo (LIM-49), São Paulo, São Paulo, Brazil

[4]Universidade de São Paulo, Faculdade de Medicina, Departamento de Infectologia e Medicina Tropical, São Paulo, São Paulo, Brazil

[5]Centers for Antimicrobial Optimization Network, São Paulo, São Paulo, Brazil

[6]Universidade Federal do Ceará, Fortaleza, Ceará, Brazil

[7]Hospital Glória D'Or, Rio de Janeiro, Rio de Janeiro, Brazil

[8]Universidade Estadual de Londrina, Londrina, Paraná, Brazil

[9]Hospital Paulistano, São Paulo, São Paulo, Brazil

[10]Hospital Unimed, Espírito Santo, Brazil

[11] Hospital Santa Catarina, São Paulo, São Paulo, Brazil

[12]Hospital do Servidor Público Estadual, São Paulo, São Paulo, Brazil

Correspondence to: Silvia Figueiredo Costa
Universidade de São Paulo, Faculdade de Medicina, Hospital das Clínicas,
Av. Dr. Enéas Carvalho de Aguiar, 255,
CEP 05403-900, São Paulo, SP, Brazil
E-mail: silviacosta@usp.br

Received: 20 October 2025
Accepted: 24 February 2026

Editor: Thelma Suely Okay

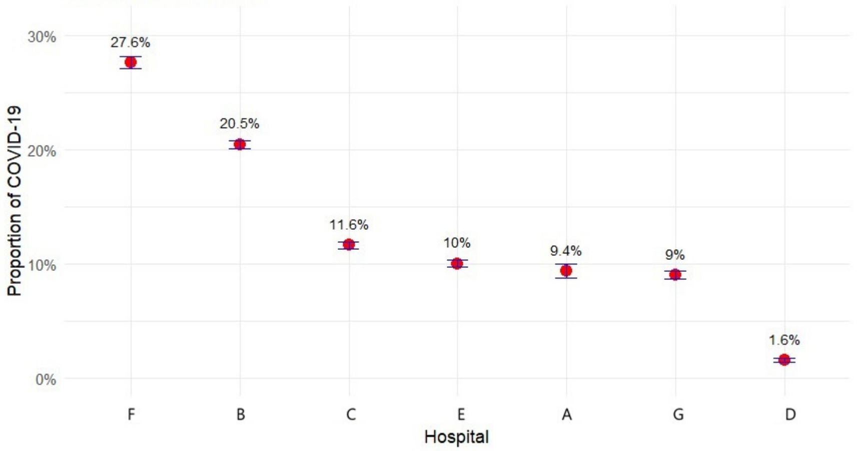

Supplementary Figure S1 - Proportion of COVID-19 admissions across seven Brazilian hospitals.

This is an open-access article distributed under the terms of the Creative Commons Attribution License.
